# Semi-supervised learning for the identification of syn-expressed genes from fused microarray and *in situ *image data

**DOI:** 10.1186/1471-2105-8-S10-S3

**Published:** 2007-12-21

**Authors:** Ivan G Costa, Roland Krause, Lennart Opitz, Alexander Schliep

**Affiliations:** 1 Department Computational Molecular Biology, Max Planck Institute for Molecular Genetics, Berlin, Germany; 2 Abteilung Entwicklungsbiochemie, Universität Göttingen, Göttingen, Germany; 3 Department Cellular Microbiology, Max Planck Institute for Infection Biology, Berlin, Germany

## Abstract

**Background:**

Gene expression measurements during the development of the fly *Drosophila melanogaster *are routinely used to find functional modules of *temporally *co-expressed genes. Complimentary large data sets of *in situ *RNA hybridization images for different stages of the fly embryo elucidate the *spatial *expression patterns.

**Results:**

Using a semi-supervised approach, constrained clustering with mixture models, we can find clusters of genes exhibiting *spatio-temporal *similarities in expression, or *syn*-expression. The temporal gene expression measurements are taken as primary data for which pairwise constraints are computed in an automated fashion from raw *in situ *images without the need for manual annotation. We investigate the influence of these pairwise constraints in the clustering and discuss the biological relevance of our results.

**Conclusion:**

Spatial information contributes to a detailed, biological meaningful analysis of temporal gene expression data. Semi-supervised learning provides a flexible, robust and efficient framework for integrating data sources of differing quality and abundance.

## Background

The study of embryonic development of the fly *Drosophila melanogaster *revealed many genes important to the development of other metazoans, including humans. Knowing the precise localization and time of gene expression is crucial in the elucidation of these dynamic cellular mechanisms. The advent of microarray technology has led to the generation of data sets measuring transcription or gene expression levels over the complete embryonic development of the fly [1-4]. Under the assumption that genes with similar expression patterns have similar properties, the concept of *co-expressed genes *can be used to generate hypotheses about function, pathways and role of proteins that can be taken to the laboratory for further investigation. This reasoning, guilt-by-association, firmly installed the use of clustering algorithms in the analysis of data from DNA microarrays [5], as a cluster of genes ideally represent functional modules. For example, proteins expressed from co-regulated gene sets have been shown to physically interact [6,7].

Time-course data collected during the cell-cycle, development and differentiation, or in response to external factors required novel methods to cope with the temporal dependencies inherent to gene expression time-courses and with data quality issues; see [8] for a recent review, which identified mixture models to be preferable. Hence, mixture models and model-based clustering found wide-spread use in gene expression time-course analysis [9-18]. The robustness of mixture models and their flexibility to capture dependencies in the data by use of Splines [9,10] or Hidden Markov Models (HMM) [14-16] as component models are the main reasons for the success of this technique in gene expression analysis. While the performance of some methods is quite impressive, the dimensionality of typical data sets – ten-thousands of genes, less than twenty time-points – implies that apparent co-expression of genes can be observed due to chance and that, hence, the value of information transfer between co-regulated genes is limited.

### Semi-supervised learning and Heterogeneous Data

**Figure 1:**
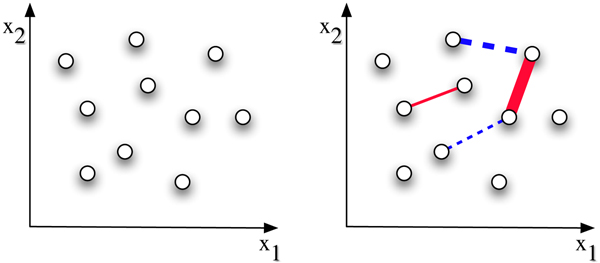
**Semi-supervised clustering**. The effectiveness of pairwise constraints is shown by contrasting with the unsupervised setting (left). Assuming a two-dimensional space, the addition of positive pairwise constraints, depicted as red edges, and negative constraints depicted as blue edges (right), can indicate existence of two or more clusters and the cluster boundaries. Edge width corresponds to constraint magnitude.

One way to strengthen the concept of co-expression for clustering algorithms is to augment the *primary *data, gene expression time-courses in our application, with *secondary*, external data in order to yield biologically more plausible solutions; recall that most clustering algorithms are only guaranteed to converge locally. The framework of choice which fits in nicely with the iterative knowledge acquisition process in biology is semi-supervised learning [19], partly clustering (unlabeled learning) and partly classification (labeled learning); sometimes this is also referred to as constrained clustering [20-22] (see Fig. 1). One of the first applications in bioinformatics [14] shows, that less than 2% labels can drastically improve clustering quality. On real data, high-quality labels which indicate whether, for example, two genes are part of the same functional module can be obtained from the literature. Use of abundant annotations from the Gene Ontology (GO) [23] that are often used to validate clusterings [24] provide surprisingly little improvement, partly due to a mismatch between the semantics of GO and similarity of expression. In this work we use a formulation proposed in [20] that can be combined with mixture model estimation.

### Spatial expression patterns

Another important aspect of gene expression, its precise localization, has been studied in great detail in the fly. While the prime motivation for these sensitive experiments was to understand the role of individual genes in organ development, we can incorporate the spatial expression patterns for the generation of functional hypotheses.

Genes that share the same temporal-spatial expression patterns are more likely to form a functional module. If they are synchronously co-expressed in *one tissue*, or in *multiple tissues *we speak of *syn-expression *[25] and take in particular the latter case as a strong sign of functional similarity. The spatial expression patterns can be determined with *in situ *experiments where an mRNA-specific stain is produced by mRNA-binding oligonucleotides and a suitable dye [26]. Further processing, imaging and image analysis produces either 2D or 3D images of spatial patterns of gene expression; large-scale data sets are available for fly development and for other model organisms. Even though *Drosophila *embryos are morphologically simple, the image analysis is quite involved as *in-situ *images are taken of many different subjects with large fluctuations in shape. In addition, the staining intensity has higher, gene-specific error rates compared to DNA microarrays.

### Prior work

Tomancak *et al*. [4] performed a large scale study of gene expression in the fly embryos by *in situ *RNA hybridizations. The images were manually curated and annotated using a controlled vocabulary – ImaGO – following the example of the Gene Ontology [23]. The final result was a hierarchical clustering of genes based on the manual annotations; the gene expression time-courses were not included in the analysis. Further work concentrated on mining the image database for genes with a spatial expression pattern similar to a query [27,28] and on the extraction of features deemed peculiar and noteworthy [28], for example by clustering images based on an eigenvector based representation [29]. Recent smaller scale studies investigated pattern formation in Drosophila based on 3D *in situ *images [30,31] for a small number of genes.

### Our contribution

**Figure 2:**
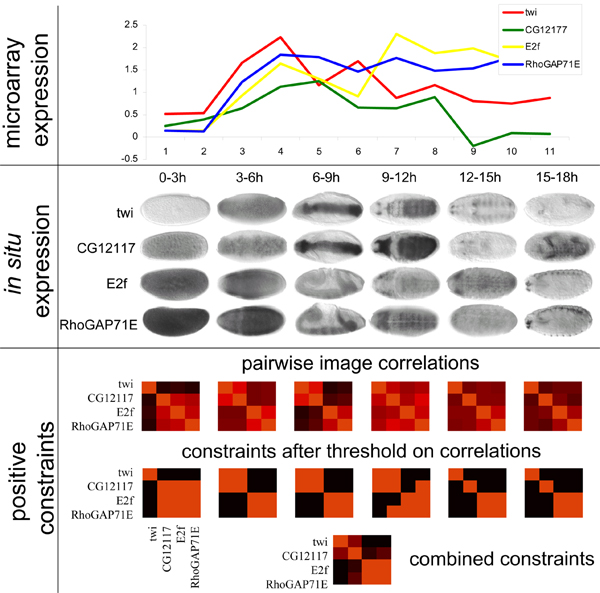
**Obtaining constraints from images**. We depict the time course expression (top) and registered *in situ *images (middle) of genes *twi*, *G12177*, *Ef2 *and *RhoGAP71E*, which indicate their temporal and spatial expression patterns. From left to right, the embryo images are categorized into the time periods 0–3 h, 3–6 h, 6–9 h, 9–12 h, 12–15 h and 15–18 h. The microarray expression displays a similar expression pattern with maximal expression after 3 hours for all genes but weakly diverging at later time points. The *in situ *images indicates that *twi *and *CG12177 *have syn-expression at time periods 3–6, 6–9 and 9–12; while *Ef2 *and *RhoGAP71E *at periods 0–3, 3–6, 6–9, 9–12 and 15–18. At the bottom, we display how positive constraints are derived from *in situ *hybridization patterns. A heat-map displays the correlation coefficients between all pairs of *in situ *images of the corresponding time period (red values indicate positive correlations). After thresholding the correlation matrices, a constraint matrix for each time period is obtained. For example, constraint matrices from periods 3–6 and 6–9 indicates syn-expression of pairs (*twi*, *CG1217*) and (*Ef2*, *RhoGAP71E*), while the constraint matrix from period 9–12 also indicate that (*CG1217*, *RhoGAP71E*) are syn-expressed. The matrices are combined into one constraining genes that display syn-expression in at least three periods, as indicated in the matrix at the bottom.

We obtain clusters of syn-expressed genes during the development of *Drosophila*. We propose to automatically infer positive constraints (spatial co-expression) and negative constraints (expression in distinct tissues) from the *in situ *image data and use them in a mixture model for the complementary, higher quality, DNA microarray time-course data as shown in Fig. 2.

## Results and discussion

### Clustering of gene expression data using mixture of multivariate Gaussians

We cluster gene expression data using a mixture of multivariate Gaussians with diagonal covariance matrix and choose the number of components to be 28 as suggested by the Bayesian Information criterion (BIC) (see Section Evaluation, [32]).

**Figure 3:**
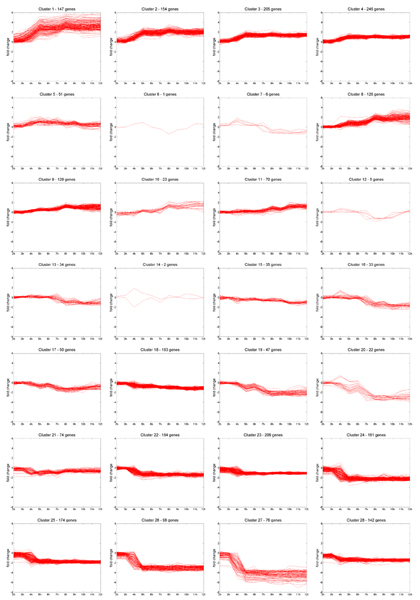
**Clustering result: Mixture of Gaussians**. The similarity of overall patterns in the clustering result of the MoG is explained by the developmental stages investigated. The major phenomena are depletion of maternal mRNA (maternal genes) and start of the embryonic transcriptional machinery during embryogenesis at time point 3 hours (zigotically expressed genes). In the clusters with zigotically expressed genes, we observe two main periods of activation: 3–4 hours for cluster U1 to U5, and 7–8 h for clusters U8 to U11. In the clusters with maternal genes, we observe under-expression of genes at several time periods: 3–4 h in clusters U21 to U28; 4–5 h for clusters U17 to U20; 6–7 h for cluster U16; 7–8 h for clusters U12 and U13; and 9–10 h for cluster U15.

The gene expression time-courses cover the period from 1 to 12 hours of the embryo development and expression values are given as log-ratios (See Section Data for details). Overall, our clustering results reflect two typical classes (see Fig. 3), the maternal and zygotic transcripts [33]. Maternal genes appear strongly expressed in the first three hours, usually followed by a decline. The clusters 18 to 28 clearly follow a maternal pattern. These transcripts are deposited in the oocyte; typically the embryo does not transcribe these genes in early development. They are responsible for the determination of body axes and the first phases of the cell cycle and other functions. The period from 2 to 3 hours coincides with the cellularization and the formation of three germ layers following gastrulation, when primary tissues start to develop [34]. Conversely, genes actively transcribed in the embryo are not expressed in the early time points and expression rises to significant levels only in later stages (3 hours and later). Many of these genes are important to organogenesis. Transcripts in the clusters 1 to 4 and 8 to 11 follow the pattern of embryonic activation unambiguously. The functional association can be observed in the overrepresented Gene Ontology terms (see Supplementary Material [35]). For other clusters, shapes cannot be matched to such simple schemes. Several have maximal expression in the midst of embryonic development. Note that the clusters that show varying levels are less populated than the ones in the maternal and in the activated class.

### Using images as Partial Information

We use semi-supervised learning to obtain better solutions for the maximum-likelihood estimation by constraining the mixture estimation with pairwise constraints between genes. The principle behind this is shown in Fig. 1. We choose a very simple approach to compare the images, which gives competitive results compared to a computationally more complex previous approach [36], combined with judicious filtering. The constraints are derived by measuring correlation between *in situ *images of pairs of genes. Pairs of genes, whose images are highly correlated in three or more time periods, are positively constrained, see Fig. 2 for example. Negative constraints are derived similarly (see Section Constraints from *in situ *data for details). These constraints will, ideally, differentiate between genes showing co-expression due to chance and causal temporal co-expression also supported by spatial co-expression (syn-expression).

As a previous study has shown [24], noisy constraints will be detrimental to the clustering quality; consequently few high quality constraints are preferable compared to many constraints of medium or low quality. The correlation coefficients of all pairwise image comparisons showed a bi-modal distribution (not shown) which allowed to select the strongest correlations with little ambiguity. Thus we arrived at a set of constraints derived from strongly positive and negative correlations.

### Changes in the biological annotations

**Figure 4:**
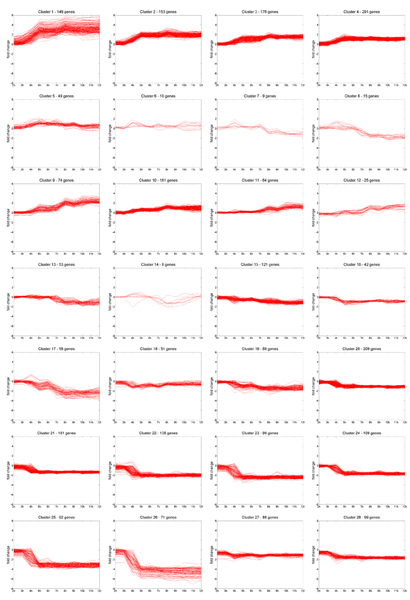
**Clustering result: Constrained Mixture of Gaussians**. The 28 clusters from cMoG show tightly co-regulated patterns and a refinement of the clustering solution of MoG. In the clusters with zygotically expressed genes, we also observe two main periods of activation: 3–4 h for clusters c1 to c5, and 7–8 h hours for clusters c9 to c12. In the clusters with maternal genes, we observe under-expression of genes at several time periods: 3–4 h for clusters C18, C20 to C28; 4–5 h for clusters C15, C16, C19; 6–7 h for clusters C8, C13, C14 and C19; and 7–8 h hours for cluster C7.

To investigate the effects of the constraints in the clustering, we compare the results of the mixture of Gaussians (MoG) against the mixture of Gaussians with pairwise constraints (cMoG) (see Fig. 4 for clusters). As explained in Section Evaluation, we choose to use positive constraints, which are supported in at least three developmental stages, as they yield good recall of *in situ *image annotations.

As a sanity check, we inspect if cMoG is successful in constraining the clustering by counting the number of constraints, as derived from the images, met in the final solutions. With MoG, a sizeable proportion of the constraints are already satisfied (656 out of 1756 pairwise positive constraints), as the expression data partially agrees with the constraints as syn-expressed genes are co-expressed. With cMoG, 1127 out of 1756 pairwise positive constraints are met, nearly twice the number for MoG. This demonstrates that cMoG benefits from the constraints in deriving the clusters.

**Figure 5:**
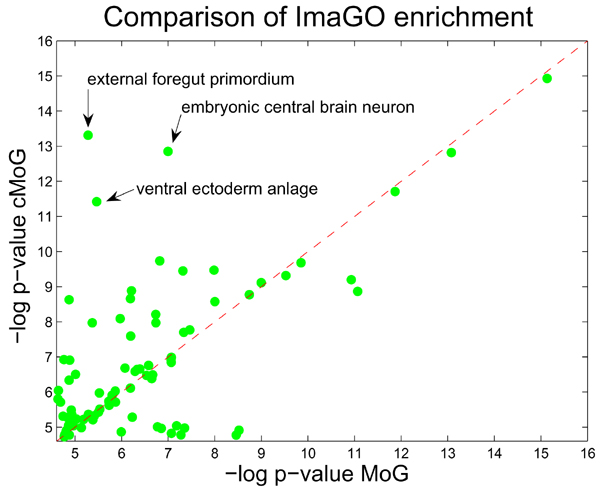
**ImaGO term enrichment**. We compare ImaGO term enrichment of MoG (*x*-axis) and cMoG (*y*-axis) in a scatter plot. We use -*log*(*p*)-values, thus larger values indicate a larger degree of enrichment. Points above the red line indicate a higher enrichment in cMoG clusters, and values below in MoG clusters. The distance from the diagonal is proportional to the increase in enrichment. For 67 out of 112 ImaGO terms we observe a higher degree of enrichment in cMoG clusters.

**Figure 6:**
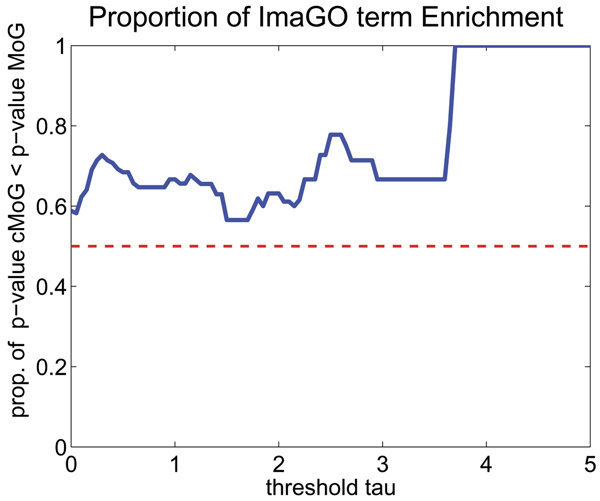
**Proportion of ImaGO term enrichment**. For each threshold *τ *(*x*-axis), we depict the proportion of ImaGO terms for which we observer a smaller *p*-value in cMoG than in MoG (*y*-axis). The threshold *τ *discards ImaGO terms, where the difference in the log of the *p*-value of cMoG and MoG in smaller then *τ*. As can be observed, the proportion is higher then 0.5 for all *τ *values, which indicates an advantage of cMoG. Furthermore, the proportion has an increasing tendency for higher *τ *values.

**Figure 7:**
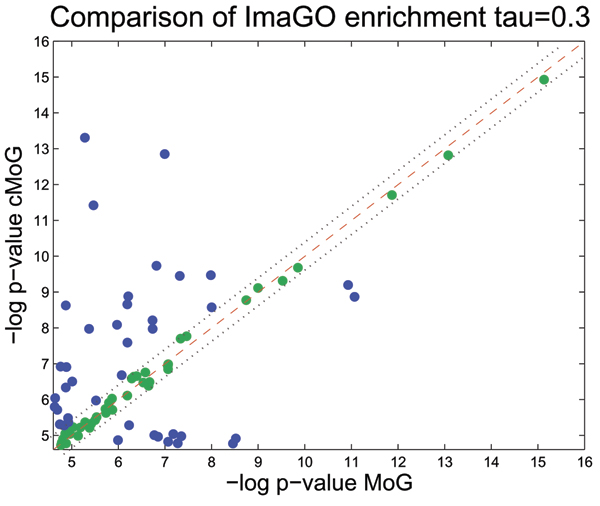
**ImaGO term enrichment for *τ *= 0.3**. We compare ImaGO term enrichment of MoG (*x*-axis) and cMoG (*y*-axis) in a scatter plot for *τ *= 0.3. We use -*log*(*p*)-values, thus larger values indicate a larger degree of enrichment. Points above the red line indicate a higher enrichment in cMoG clusters, and values below in MoG clusters. Green points between the dotted lines represent ImaGO terms not satisfying the threshold *τ *= 0.3, where *τ *indicates the distance from the diagonal line to the dotted lines. We clearly observe a higher proportion of non-filtered ImaGO terms (points in blue) above the diagonal line (32 ImaGO terms) against (12 ImaGO terms) below the diagonal. A binomial test is rejected with a *p*-value of 0.0018, which indicates an significant advantage of cMoG.

Another helpful analysis is the comparison of enrichment of *in situ *image annotations (ImaGO), as described in Section Evaluation (see [35] for complete results). We display in Fig. 5 a scatter plot with the *p*-values of all ImaGO terms, which had an enrichment *p*-value below 0.01 in one either cMoG or MoG clusters. In summary, cMoG has a higher enrichment in 67 out of 112 relevant ImaGO terms. A binomial test for testing the event of having 67 successes in 112 trials is rejected with a *p*-value of 0.0232, which indicates that the counts of ImaGO terms with higher enrichment for cMoG is significantly higher than expected by chance. Furthermore, if we take only ImaGO terms with a higher enrichment gain for one of the methods into account (points distant from the diagonal line in Fig. 5), the advantage of cMoG is even greater (see Fig. 6 and Fig. 7). This indicates that even without direct use of the annotation information from ImaGO, cMoG has a greater sensitivity in grouping syn-expressed genes.

Overall, the individual clusters of MoG and cMoG did not differ much; the cMoG clusters were better spread and the number of clusters with few genes assigned is smaller. One way to quantify the distinctions is to calculate the sensitivity and specificity of cMoG taking the results from MoG as the ground truth. These values are respectively 0.53 and 0.97, which indicates that cMoG has a tendency to subdivide clusters from MoG.

### Functional annotations in constrained clusters

Even for a well characterized genome like *Drosophila *the high dimensionality in the annotation data provides only limited information for any single gene. Analyzing the obtained clusters is also challenging due to the necessity to identify the corresponding functional modules in the unconstrained and the constrained sets and by the requirement to show improvements rather than simple correct functional assignments. In the following, we will refer to the *i*th cluster from cMoG and MoG as C*i *and U*i *respectively. For some cases, the mapping from clusters of cMoG to MoG is simply one to one (e.g., C1 to U1, C5 to U5, C11 to U11 and C12 to U10). Most other clusters show larger differences. We focus our functional analysis on clusters with zygoticly expressed transcripts (i.e., C1 to C4 and C9 to C12 in Fig. 4).

**Figure 8:**
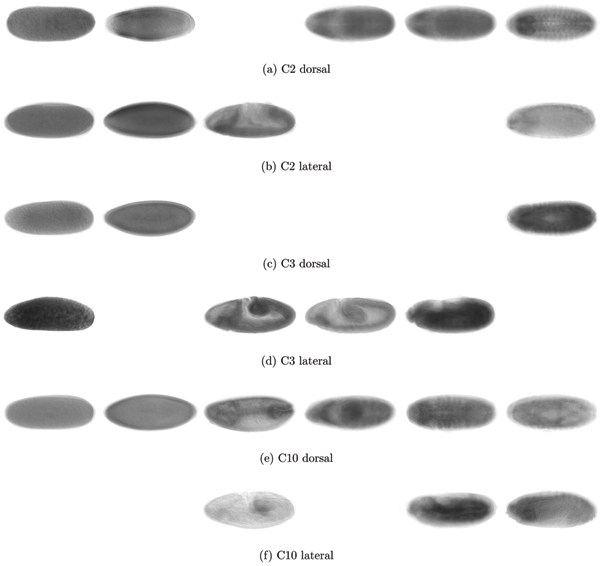
**Averaged *in situ *images C2, C3 and C10**. Averaged *in situ *images of genes constrained in Cluster C2 (top), C3 (middle) and C10 (bottom) allow to visually assess homogeneity of spatial distribution. From left to right, we have embryos at hours 0–3, 3–6, 6–9, 9–12, 12–15 and 15–18. Top images represents dorsal views, bottom images lateral views; not all time periods have images in both views.

Cluster C2 represents a good example of the changes resulting from the introduction of constraints. It contains most of the genes from U2 (135 genes) and 16 genes from U3. Out of the seven genes, which show similar expression patterns and have co-location constraints (*CG6930*, *E2f*, *Iswi*, *neur*, *Set*, *RhoGAP771e*, *trx*), only four (*G6930*, *E2f*, *Iswi*, *trx*) are found in the U2. All these genes have ImaGO annotations related to *ventral nerve cord primordium *and related terms (see Fig. 8 top for mean *in situ *images of these genes and [35] for complete ImaGO enrichment results). Related genes that have no constraints but are annotated as part of the *embryonic central nervous system *are included in C2 (*CG7372*, *CG14722*, *fzy*). The analysis of GO term enrichment indicates terms such as *nervous system development *(*p*-value of 3.38e-23) and *system development *(*p*-value of 9.54e-21) (similar term enrichment is found for cluster U2). It should be noted that the clusters U2 and U3 are similar overall and mainly differ in the average time when genes reach the plateau of maximal expression.

An example for larger changes is cluster C3, which is mainly composed of genes originally found in U3 (101 genes) and U8 (63 genes). C3 was constrained by three genes (*rhea*, *Rsf1 *and *vig*) of which *rhea *and *vig *come from cluster U8 and *Rsf1 *from U3 (see Fig. 8 middle for mean *in situ *images of C3). This cluster presents smaller *p*-values for ImaGO terms related to *muscle primordium *(genes *CG5522*, *CG9253*, *Dg*, *Mef2*, *betaTub60D*, *htl*, *mbc*, *vig*) than U3 and U8. Furthermore, GO term analysis reveals that this cluster shows enrichment for *nervous system development *(*p*-value of 1.33e-11) and *axis specification *(9.31e-05). For the latter term, seven genes are originally from U3 (*Dfd*, *Lis-1*, *sti*, *Syx1A*, *sqd*, *Ras85Dm*, *tup*) and five from U8 (*baz*, *Dg*, *pnt*, *Rac2*, *tok*), demonstrating that the changes introduced increased the number of syn-expressed genes within C3.

The cluster C9 represents only a subset of U8 (59 out of the 126 genes) but has no genes with constraints. It consists of genes from U8 that are not constrained to genes from C3 (see paragraph above). Still, it is enriched in the ImaGO term *embryonic central nervous system *and related terms (genes *HLHmbeta*, *NetB*, *Oli*, *lin-28*, *scrt*, *sd*, *tap*, *uzip *and *zfh2*). The cluster is also enriched in the terms *organ *(*p*-value 2.66e-05) and *ectoderm development *(*p*-values 8.54e-05), which were significantly enriched in U8. In other words, this cluster is a specialization of U8, whose genes are specific to *organ development*.

C10 is formed by the addition of most genes in the U4 cluster (39 genes) to U10 (118 genes). There are seven genes constraining this cluster (*CG6751*, *CG18446*, *CG13912*, *CG10924*, *CG8745*, *dm*, *Klp61F*) (see Fig. 8 bottom). ImaGO term enrichment relates this cluster to *yolk nuclei *and *amnioserosa*. It is also in enriched in the GO term *nervous system development *(*p*-value 1.06e-08), all of which were insignificant in the U10 cluster.

It is also worthwhile to look at those few cases where MoG performed better. From Fig. 5, two ImaGO terms with higher enrichment increase in MoG are *maternal *and *procephalic ectoderm anlage in statu nascendi*. The first term was enriched in cluster C22 and U21, where MoG had some more genes related to the term *maternal *(34 genes in MoG compared to 31 genes in cMoG). For the latter ImaGO term, clusters U2 and C2 were both enriched, and there was only one annotated gene in U2 not in C2. As none of these annotated groups of genes had pairwise constraints, we could not detect any direct effect of the constrained clustering on these results.

The refined clusters improve the generation of testable hypotheses for the role of uncharacterized genes. Overall, we observe improvement in annotation of genes related to development of the fly, in particular with respect to the ImaGO annotations, which increases our confidence in the delineation of syn-expressed functional modules.

## Conclusion

The generation of functional hypotheses by integrating different information sources is a key problem posed by the massive amounts of high-throughput data that is generated in todays laboratories. Often, analyses are limited to few information sources and the integration only starts after many processing steps, frequently including manual annotation, and is more often than not performed manually.

Here we have shown, for a limited setting, how to automatically fuse temporal and spatial gene expression patterns by semi-supervised clustering. Our results show that the clusters we find are biologically meaningful and that we can detect clusters of syn-expressed genes which are worthwhile targets for further investigation, either with classical biological analysis or as the input for methods inferring networks. Our implementation is reasonably simple and computationally efficient and the semi-supervised approach provides a flexible framework for adapting results to questions biologists are interested in. The main advantage of the semi-supervised approach over joint models and other approaches is that it can cope easily with the variations in data abundance from different sources. Gene expression measured with DNA microarrays is often available for ten-thousands of genes and many time-points, in situ hybridization will typically only cover a fraction of those, as will high quality protein interaction or protein structure data. Our results show a small but very clear improvement, despite the complexity of the problem, namely the restriction to embryonic stages to few data points, and the usual caveats concerning DNA microarrays and *in situ *images of gene expression. The open questions are manifold and concern both the biology and the computer science. How can we refine biological questions to yield more meaningful answers? How can one mine image data effectively, and does a representation in 2D suffice or is using the third dimension a necessity? The best combination of several, potentially conflicting, information sources to arrive at one set of constraints is an equally challenging problem. Can one incorporate the per-stage constraints into the learning for time-course data? We have demonstrated that our methodology is a promising candidate for the delineation of functional modules using different data types and our results show that further investigations are likely to bear fruit.

## Methods

### Mixture models

A mixture model [32] is a stochastic model where observations are drawn from one of several component densities. More formally, it is a convex combination of density functions,

$$P[x_{i}|\Theta ]=\sum_{k=1}^{K}\alpha_{k}P[x_{i}|\theta_{k}].$$

Here, $X={\left\{x_{i}\right\}}_{i=1}^{N}$ denotes the observed data (or the gene expression time-courses), Θ = (*α*_1_,...,*α*_*K*_, *θ*_1_,...,*θ*_*K*_) the non-negative component weights or priors *α*_*k*_, *i *= 1,...,*K*, which add to unity and **P**[*x*_*i*_|*θ*_*k*_] are the *K *component densities parameterized by *θ*_*k*_, *k *= 1,...,*K*, for example *θ*_*k *_= (*μ*_*k*_, Σ_*k*_) for multivariate Gaussians. The Expectation-Maximization (EM) algorithm [32] can be used to find parameters Θ* maximizing (1) at least locally. The EM is necessary as (1) is essentially the incomplete data likelihood function; missing are the values of the indicator variables $Y={\left\{y_{i}\right\}}_{i=1}^{N}$, *y*_*i *_∈ {1,...,*K*}, which designate the component *y*_*i *_which generated the observation *x*_*i*_. If *Y *is known, the maximization is straight-forward, and the core idea of EM is to iteratively use expected values for *Y *based on current parameters Θ_*t *_in the estimation of Θ_*t*+1_. A nice introduction to the EM-algorithm is given in [37].

### Partially supervised learning

We assume additional soft constraints for observations in the form of pairwise positive (link) respectively negative (do not link) constraints $w_{ij}^{+}$ respectively $w_{ij}^{-}$ ∈ [0, 1], which reflect the degree of linking for each pairs of observations *x*_*i*_, *x*_*j*_, 1 ≤ *i *<*j *≤ *N*. We use a formulation proposed by Lange et al. [20] which we summarize here; for further applications of the method we refer to [21,24].

Let *W*^+ ^= {$w_{ij}^{+}$} respectively *W*^- ^be symmetric *N *× *N *matrices. The EM-algorithm can be easily modified to respect the constraints *W*^+^, *W*^-^. In the *t*-th E-step the posterior distribution *P*[*Y*|*X*, Θ_*t*_] over hidden labels *y*_*i *_is computed, where Θ_*t *_is the last estimate of the parameters. By Bayes' rule we have

$$P[Y|X,\Theta ]=\frac{1}{Z}\cdot P[X|Y,\Theta ]\cdot P[Y|\Theta ],$$

where *Z *is a normalizing constant. Loosely speaking, the constraints are incorporated by choosing the prior distribution **P**[*Y*|Θ_*t*_] such that neither constraints *W*^+^, *W*^- ^nor prior probabilities *α*_*k *_in Θ_*t *_are violated while maximizing its entropy. In other words, we choose the distribution, which obeys the *maximum entropy *principle and which is called the *Gibbs *distribution. See [20,21] for full details. Hence

$$P[Y|\Theta ]=\frac{1}{Z}\prod_{i}\alpha_{y_{i}}\prod_{i,j}\exp(-\lambda^{+}w_{ij}^{+}(1-\delta_{y_{i}y_{j}})-\lambda^{-}w_{ij}^{-}\delta_{y_{i}y_{j}}),$$

where *Z *is the normalizing constant. The Lagrange parameters *λ*^+ ^and *λ*^- ^weigh the penalty of positive and negative constraints violations and hence control the importance of the constraints. If *λ*^+ ^= *λ*^- ^= 0 then the estimation maximized the likelihood, whereas for increasing *λ*^+^, *λ*^- ^the result is more strongly influenced by the constraints. As computing (2) is usually infeasible we again follow [20] and resort to a *mean field approximation*. Note, finally, that when there is no overlap in the constraints – more exactly, $w_{ij}^{+}w_{ij}^{-}=0$, and *λ*^+ ^= *λ*^- ^~ ∞ – we obtain hard constraints [16,38].

### Data

We use the data-set described in [39] which is available from the BDGP database [40].

#### Image collection

Embryos of Canton were collected and aged to produce embryos 0–3, 3–6, 6–9, 9–12, 12–15 and 15–18 hours old. The *in situ *reactions were based on a cDNA library of 2,721 clones; in the end images were collected for 1,388 genes. The difference is caused either by a failure of *in situ *reactions or by a lack of tissue-specific expression. Images were taken with a dissecting microscope in different focal planes and different orientations.

#### Time-courses

For twelve consecutive one-hour time windows of embryogenesis mRNA levels were measured using the Affymetrix GeneChip *Drosophila *Genome array targeting about 14,000 genes and processed with the standard Affymetrix tool suite. We used the median from three biological replicates. As the embryos were not synchronized, the manual inspection of the morphology was used to establish a common time-scale with the time-course data. Expression values were transformed to log-ratios by using time point 1 hour as reference. We removed genes not exhibiting at least a two-fold change, which leaves us with 2684 genes.

### *In situ *image processing

The majority of *in situ *hybridization images in the BDGP database [40] contain the projection of exactly one centered embryo. However, there is a noticeable portion of images with multiple touching embryos. To exploit as much data as possible, the goal of image preprocessing is to locate and extract exactly one complete embryo from each image, even for touching embryos.

To distinguish between embryo and non-embryo pixels we estimate the local variance of grey level intensities for each pixel in a 3 × 3 neighborhood, following [27]. It suffices to apply a fixed predefined threshold for segmentation using variance estimates because of a homogeneous background in contrast to the embryo. To eliminate erroneous embryo regions a sequence of morphological closing and opening using a circular mask of radius 4 is applied [41]. Subsequently the largest connected component is extracted. The resulting region may be the projection of a single complete or partial embryo or the projection of a set of multiple touching embryos. To distinguish these different cases we apply a series of simple filters based on ellipticity, compactness and area of the extracted region. For regions of multiple touching embryos we introduce a procedure to separate the individuals and to extract a single complete high quality embryo. Further details are given in [36].

**Figure 9:**
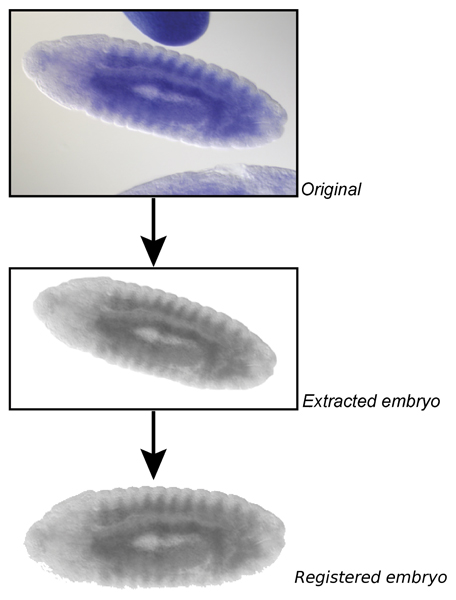
**Image processing pipeline**. The image pipeline combines registration, morphological operations and further processing steps to automatically process raw images, even if they include multiple touching embryos. Shown here is the image insitu8784, gene *CG5353*.

The final step of image processing is to register the embryos extracted to a standardized orientation and size to allow for comparison of different expression patterns. The embryo is rotated to align horizontally to the principal axis. Subsequently the bounding box is scaled to a standard size. Fig. 9 shows the steps of the image processing pipeline for one example image.

### Constraints from *in situ *images

To compare *in situ *hybridization patterns between a pair of registered embryo images, we compute the Pearson correlation as a co-location index, as proposed in [36]. This index takes both the spatial distribution and the strength of hybridization into account. Despite its simplicity, it had similar performance, in a querying scenario, compared to more complex methods such as the one proposed in [27]. More formally, let *X *and *Y *describe the pixel intensities of two equal sized and registered embryo images, the Pearson correlation is calculated as

$$CC(X,Y)=\frac{Cov(X,Y)}{\sqrt{Var(X)}\sqrt{Var(Y)}}.$$

The 18 developmental stages of the embryo are divided into six periods (0–3, 3–6, 6–9, 9–12, 12–15 and 15–18). Not all time points were sampled for each gene and for some periods and genes, *in situ *images were taken in a dorsal and/or lateral views. There is however no annotation of the orientation of the embryo; automatic registration being a difficult task for this problem. Hence, for each pair of images, we estimate the correlation between all possible orientations and take the maximum value. For a pair of genes and a developmental period, we repeat the above procedure for all pairs of images and again keep the maximum value. By an inspection of the distribution of the correlation coefficient, we select a value *k *of gene pairs to constrain. In other words, the gene pairs (*g*_*i*_, *g*_*j*_) displaying the *k*th highest correlations are positively constrained ($w_{ij}^{+}$ = 1). Similarly, the gene pairs (*g*_*i*_, *g*_*j*_) displaying the *k*th lowest correlations are negatively constrained ($w_{ij}^{-}$ = 1). As we are interested in high quality constraints, we use conservative thresholds, which select only a small percentage of gene pairs to be constrained (less then 2% of genes with *in situ *images). As a last step, we need to combine the image constraints from the distinct developmental periods. Again, we use a conservative strategy, requiring that a pair of genes is only constrained if we observe a correlation coefficient exceeding our threshold in at least three respectively four developmental periods; cf. Fig. 2 for an example. With support of at least three periods, there are 1,756 positive constraints within 170 genes and 2,544 negative constraints within 360 genes. With support of at least four stages, there are 270 positive constraints within 66 genes and 640 negative constraints within 151 genes.

### Evaluation

We use multivariate Gaussians with diagonal covariance matrices [32] as our components in all mixture estimations, as we are mainly interested in comparing our semi-supervised approach with the unsupervised scenario. For a given mixture parameterization, we initialize models randomly, repeat the estimation 15 times and choose the one with maximum likelihood. We estimate the optimal number of clusters with the Bayesian Information Criteria (BIC) in the unsupervised setting, which indicates 28 clusters. We use this number for all other runs described below. All data sets and a tool implementing the method are available in our Supplementary Material Web page at , where we also display plots of the clusters, lists of genes, images constraining the clusters, GO term enrichment (as provided by GoStat [42]) and ImaGO term enrichment.

#### ImaGO term enrichment

A controlled vocabulary, which follows the Gene Ontology [23] standard, was used to annotate gene expression patterns [39]. All images deposited in BDGP are annotated to at least one of these terms. We can, as usual with Gene Ontology [42], use a statistical tests to list ImaGO terms, which are overrepresented in a cluster. Given a set of *n *genes, we count the number *c *of genes in a given cluster, the number *t *of genes annotated with a given ImaGO term and the number *h *of genes that are both in the cluster and annotated with the ImaGO term. The resulting *p*-value, calculated with the Fisher Exact Test [43] is then used to assess the significance of the count *h*, given *n*, *c *and *t*. The Fisher Exact Test assumes that the data comes from a Hyper-geometric distribution, and it is equivalent to the Hyper-geometric test. Lower *p*-values indicate an enrichment in ImaGO terms and, consequently, better results.

This strategy is useful for evaluating the biological quality of a single cluster, but gives no global assessment for comparing the results given by two clustering solutions. One heuristic way to perform such an analysis is to compare the *p*-values obtained in two solutions [44]. A superior method has a larger number of ImaGO terms with lower *p*-values.

#### Selection of constraints and parameters

We evaluate the use of constraints shared by either three or four developmental periods, the use of positive constraints and both positive and negative constraints, and four choices of the parameter *λ*^+ ^= *λ*^- ^(0.5, 1.0, 1.5 and 2.0). There is no theory guiding choices of *λ*^+ ^and *λ*^-^, neither is there a definitive "gold standard" data set to optimize them. Hence, motivated by [24], we made the simple choice to give positive and negative constraints equal weight, which should have some impact on the clustering result, but not dominate it.

As shown in Table 1, all constraint combinations lead to an increase in ImaGO term enrichment, except the use of positive and negative constraints from three stages. Furthermore, values of *λ *around 1 lead to an improvement, while higher values tend to deteriorate results. Thus, we choose to use positive constraints derived from three developmental periods and a constraint weight of *λ*_+ _= 1.0 in agreement with [24].

**Table 1:** Selection of Constraints.

		Proportion of terms with lower *p*-values	
*λ*_+_	*λ*_-_	# stages ≥ 3	≥4
0.5	-	51%	48%
1.0	-	**60**%	**56**%
1.5	-	57%	49%
2.0	-	43%	46%

0.5	0.5	**49**%	44%
1.0	1.0	49%	52%
1.5	1.5	40%	**59**%
2.0	2.0	43%	47%

We show the proportion of ImaGO terms with lower *p*-values in cMoG compared to MoG for constraints from at least 3 or 4 stages, distinct constraint weights *λ*_+ _and *λ*_- _using positive constraints only respectively positive and negative constraints. Values exceeding 50% indicate an advantage of cMoG.

## Competing interests

The authors declare that they have no competing interests.

## Authors' contributions

IC implemented the approach and performed the experiments. IC and RK evaluated the clustering results. LO processed the images and wrote the parts of the manuscript describing the image processing. AS designed this study. IC, RK and AS wrote the manuscript. All authors read and approved the final manuscript.

## References

[B1] Arbeitman MN, Furlong EE, Imam F, Johnson E, Null BH, Baker BS, Krasnow MA, Scott MP, Davis RW, White KP (2002). Gene expression during the life cycle of Drosophila melanogaster. Science.

[B2] Hooper SD, Boué S, Krause R, Jensen LJ, Mason CE, Ghanim M, White KP, Furlong EE, Bork P (2007). Identification of tightly regulated groups of genes during Drosophila melanogaster embryogenesis. Mol Syst Biol.

[B3] Stolc V, Gauhar Z, Mason C, Halasz G, van Batenburg MF, Rifkin SA, Hua S, Herreman T, Tongprasit W, Barbano PE, Bussemaker HJ, White KP (2004). A gene expression map for the euchromatic genome of Drosophila melanogaster. Science.

[B4] Tomancak P, Beaton A, Weiszmann R, Kwan E, Shu S, Lewis SE, Richards S, Ashburner M, Hartenstein V, Celniker SE, Rubin GM (2002). Systematic determination of patterns of gene expression during Drosophila embryogenesis. Genome Biol.

[B5] Eisen MB, Spellman PT, Brown PO, Botstein D (1998). Cluster analysis and display of genome-wide expression patterns. Proc Natl Acad Sci USA.

[B6] Ge H, Liu Z, Church GM, Vidal M (2001). Correlation between transcriptome and interactome mapping data from Saccharomyces cerevisiae. Nat Genet.

[B7] Lee I, Date SV, Adai AT, Marcotte EM (2004). A probabilistic functional network of yeast genes. Science.

[B8] Bar-Joseph Z (2004). Analyzing time series gene expression data. Bioinformatics.

[B9] Bar-Joseph Z, Gerber GK, Gifford DK, Jaakkola TS, Simon I (2003). Continuous representations of time-series gene expression data. J Comput Biol.

[B10] Luan Y, Li H (2003). Clustering of time-course gene expression data using a mixed-effects model with B-splines. Bioinformatics.

[B11] McLachlan GJ, Bean RW, Peel D (2002). A mixture model-based approach to the clustering of microarray expression data. Bioinformatics.

[B12] Medvedovic M, Yeung K, Bumgarner R (2004). Bayesian mixture model based clustering of replicated microarray data. Bioinformatics.

[B13] Ng SK, McLachlan GJ, Wang K, Ben-Tovim Jones L, Ng SW (2006). A Mixture model with random-effects components for clustering correlated gene-expression profiles. Bioinformatics.

[B14] Schliep A, Schönhuth A, Steinhoff C (2003). Using hidden Markov models to analyze gene expression time course data. Bioinformatics.

[B15] Schliep A, Steinhoff C, Schonhuth A (2004). Robust inference of groups in gene expression time-courses using mixtures of HMMs. Bioinformatics.

[B16] Schliep A, Costa IG, Steinhoff C, Schonhuth A (2005). Analyzing Gene Expression Time-Courses. IEEE/ACM Trans Comput Biol Bioinform.

[B17] Yeung KY, Fraley C, Murua A, Raftery AE, Ruzzo WL (2001). Model-based clustering and data transformations for gene expression data. Bioinformatics.

[B18] Yeung KY, Medvedovic M, Bumgarner RE (2003). Clustering gene-expression data with repeated measurements. Genome Biol.

[B19] Chapelle O, Schoelkopf B, Zien A, (Eds) (2006). Semi-Supervised Learning.

[B20] Lange T, Law MHC, Jain AK, Buhmann JM (2005). Learning with Constrained and Unlabelled Data. CVPR (1).

[B21] Lu Z, Leen T, Saul LK, Weiss Y, Bottou L (2005). Semi-supervised Learning with Penalized Probabilistic Clustering. Advances in Neural Information Processing Systems.

[B22] Xing EP, Ng AY, Jordan MI, Russell S, S Becker ST, Obermayer K (2003). Distance Metric Learning with Application to Clustering with Side-Information. Advances in Neural Information Processing Systems.

[B23] Ashburner M, Ball CA, Blake JA, Botstein D, Butler H, Cherry JM, Davis AP, Dolinski K, Dwight SS, Eppig JT, Harris MA, Hill DP, Issel-Tarver L, Kasarskis A, Lewis S, Matese JC, Richardson JE, Ringwald M, Rubin GM, Sherlock G (2000). Gene ontology: tool for the unification of biology. The Gene Ontology Consortium. Nat Genet.

[B24] Costa I, Schliep A (2006). On the feasibility of Heterogeneous Analysis of Large Scale Biological Data. Proceedings of ECML/PKDD 2006 Workshop on Data and Text Mining for Integrative Biology.

[B25] Niehrs C, Pollet N (1999). Synexpression groups in eukaryotes. Nature.

[B26] Tautz D, Pfeifle C (1989). A non-radioactive in situ hybridization method for the localization of specific RNAs in Drosophila embryos reveals translational control of the segmentation gene hunchback. Chromosoma.

[B27] Peng H, Myers EW, Bourne PE, Gusfield D (2004). Comparing in situ mRNA expression patterns of Drosophila embryos. RECOMB.

[B28] Pan JY, Guilherme A, Balan R, Xing EP, Traina AJM, Faloutsos C (2006). Automatic mining of fruit fly embryo images. KDD'06: Proceedings of the 12th ACM SIGKDD international conference on Knowledge discovery and data mining.

[B29] Peng H, Long F, Eisen MB, Myers EW (2006). Clustering gene expression patterns of fly embryos. ISBI.

[B30] Hendriks CLL, Keränen SVE, Fowlkes CC, Simirenko L, Weber GH, DePace AH, Henriquez C, Kaszuba DW, Hamann B, Eisen MB, Malik J, Sudar D, Biggin MD, Knowles DW (2006). Three-dimensional morphology and gene expression in the Drosophila blastoderm at cellular resolution I: data acquisition pipeline. Genome Biol.

[B31] Keränen SVE, Fowlkes CC, Hendriks CLL, Sudar D, Knowles DW, Malik J, Biggin MD (2006). Three-dimensional morphology and gene expressionin the Drosophila blastoderm at cellular resolution II:dynamics. Genome Biol.

[B32] McLachlan G, Peel D (2000). Finite Mixture Models Wiley Series in Probability and Statistics.

[B33] Edgar B (1995). Diversification of cell cycle controls in developing embryos. Curr Opin Cell Biol.

[B34] Leptin M (1999). Gastrulation in Drosophila: the logic and the cellular mechanisms. The EMBO Journal.

[B35] Supplementary Material. http://algorithmics.molgen.mpg.de/Supplements/Insitu/.

[B36] Opitz L, Schliep A, Posch S (2006). Analysis of fused in-situ hybridization and gene expression data. Advances in Data Analysis.

[B37] Bilmes J (1997). A Gentle Tutorial on the EM Algorithm and its Application to Parameter Estimation for Gaussian Mixture and Hidden Markov Models. Tech rep.

[B38] Pan W (2006). Incorporating gene functions as priors in model-based clustering of microarray gene expression data. Bioinformatics.

[B39] Tomancak P, Beaton A, Weiszmann R, Kwan E, Shu S, Lewis E, Richards S, Ashburner M, Hartenstein V, Celniker S, Rubin G (2002). Systematic determination of patterns of gene expression during Drosophila embryogenesis. Genome Biology.

[B40] BDGP: Berkeley Drosophila Genome Project. http://www.fruitfly.org.

[B41] Gonzalez R, Wintz P (1991). Digital image processing.

[B42] Beissbarth T, Speed TP (2004). GOstat: find statistically overrepresented Gene Ontologies within a group of genes. Bioinformatics.

[B43] Sokal R, Rohlf F (1995). Biometry.

[B44] Ernst J, Nau GJ, Bar-Joseph Z (2005). Clustering short time series gene expression data. Bioinformatics.

